# Supplementary material for: Association between Anti-Hepatitis C Viral Intervention Therapy and Risk of Sjögren’s Syndrome: A National Retrospective Analysis
Source: J Clin Med. 2022 Jul 22;11(15):4259. doi: 10.3390/jcm11154259 (PMC9332495; doi:10.3390/jcm11154259)
Supplement: Supplementary file 1 [file jcm-11-04259-s001.zip › Supplementary table S1.pdf]

Supplementary Table S1. The corresponding ICD-9-CM codes for the diagnoses of diseases and Anatomical Therapeutic Chemical (ATC) for drug

Disease	Corresponding ICD-9-CM codes
Hepatitis C virus infection	070.41, 070.44, 070.51, 070.54, V02.62
Human immunodeficiency virus	042, 043, 044, V08, 795.8
Autoimmune rheumatic diseases	710.4, 710.1, 710.3, 710.4, 446.0–446.7, 136.1
Sjögren's syndrome	710.2
Thyroid diseases	240–242, 244–246
**Drug**	**ATC code**
Anti-HCV therapy (Interferon-based therapy)	L03AB01, L03AB02, L03AB03, L03AB04, L03AB05, L03AB06, L03AB07, L03AB08, L03AB09, L03AB10, L03AB11, L03AB12, L03AB13, L03AB14, L03AB15, L03AB60, L03AB61
