# Supplementary material for: Association between Anti-Hepatitis C Viral Intervention Therapy and Risk of Sjögren’s Syndrome: A National Retrospective Analysis
Source: J Clin Med. 2022 Jul 22;11(15):4259. doi: 10.3390/jcm11154259 (PMC9332495; doi:10.3390/jcm11154259)
Supplement: Supplementary file 1 [file jcm-11-04259-s001.zip › Supplementary table S2.pdf]

Supplementary Table S2. Hazard ratios (HRs) for Sjögren's syndrome in the propensity score-matched HCV cohort

	Crude			Adjusted*		
Variable	HR	95% CI	*p*-value	HR	95% CI	*p*-value
HCV cohort						
Untreated	1	Reference		1	Reference	
Treated with anti-HCV therapy	0.93	(0.64-1.33)	0.68	0.93	(0.65-1.35)	0.72
Sex (men/women)	0.30	(0.21-0.41)	<0.001	0.34	(0.25-0.48)	<0.001
Age (per year)	1.02	(1.01-1.03)	0.002	1.01	(0.99-1.02	0.45
Comorbidity (yes/no)						
Thyroid disease	2.43	(1.68-3.51)	<0.001	1.61	(1.10-2.34)	0.013
Geographic region						
Northern	1	Reference		1	Reference	
Central	1.74	(1.25-2.44)	0.001	2.50	(1.69-3.70)	<0.001
Eastern	0.21	(0.03-1.51)	0.12	0.35	(0.05-2.54)	0.30
Southern	0.46	(0.30-0.72)	0.001	0.58	(0.36-0.92)	0.02
Urbanization level						
Urban	1	Reference		1	Reference	
Suburban	0.71	(0.49-1.01)	0.06	0.65	(0.44-0.95)	0.03
Rural	0.72	(0.49-1.06)	0.10	0.48	(0.30-0.76)	0.002
Number of medical visits	1.01	(1.01-1.02)	<0.001	1.01	(1.01-1.02)	<0.001

Abbreviations: HCV, hepatitis C virus; HR, hazard ratio; CI, confidence interval.

*Adjusted for age per year, sex, comorbidity, geographic region, urbanization level, and number of medical visits.
